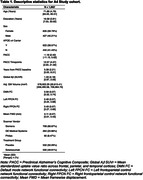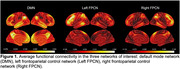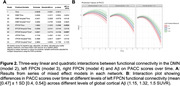# Left frontoparietal control network connectivity moderates the effect of amyloid on cognitive decline in preclinical Alzheimer’s disease

**DOI:** 10.1002/alz.090828

**Published:** 2025-01-09

**Authors:** Rory Boyle, Zahra Shirzadi, Gillian T Coughlan, Mabel Seto, Michael J Properzi, Hannah M Klinger, Diana L Townsend, Ziwen Yuan, Catherine E Scanlon, Roos J Jutten, Kathryn V Papp, Rebecca E Amariglio, Keith A Johnson, Dorene M Rentz, Jasmeer P. Chhatwal, Reisa A Sperling, Aaron P Schultz, Rachel F Buckley, The A4 Study Team

**Affiliations:** ^1^ Massachusetts General Hospital, Harvard Medical School, Boston, MA USA; ^2^ Center for Alzheimer Research and Treatment, Brigham and Women’s Hospital, Harvard Medical School, Boston, MA USA; ^3^ Brigham and Women's Hospital, Boston, MA USA; ^4^ Center for Alzheimer’s Research and Treatment, Brigham and Women’s Hospital/Harvard Medical School, Boston, MA USA; ^5^ Melbourne School of Psychological Sciences, University of Melbourne, Melbourne, VIC Australia

## Abstract

**Background:**

Stronger default mode (DMN) and bilateral frontoparietal control network (FPCN) resting‐state functional connectivity are associated with reduced β‐amyloid (Aβ)‐related cognitive decline in cognitively unimpaired older adults, who were predominantly Aβ negative. This suggests that these networks might support cognitive resilience in the face of early AD pathology but it remains unclear whether these effects are apparent in preclinical AD. We investigated whether left‐FPCN, right‐FPCN, and DMN connectivity moderated the effect of Aβ on cognitive decline using a large multi‐site dataset from the Anti‐Amyloid Treatment in Asymptomatic Alzheimer’s Disease (A4) study.

**Method:**

Cognitively unimpaired participants with elevated Aβ on screening amyloid‐PET (n=1,062, mean age=71.9 years, 60% women, see Table 1), underwent functional MRI (3T) at baseline. We obtained connectivity estimates of the left‐FPCN, right‐FPCN, and DMN using template‐based rotation (Figure 1). Cognition was measured using the Preclinical Alzheimer’s Cognitive Composite (PACC; average follow‐up including open‐label extension period =280 weeks [SD=115]). We examined three‐way linear and quadratic interactions between functional connectivity and Aβ burden with time in mixed effects models (model 2 = DMN, 3 = left‐FPCN, 4 = right‐FPCN).

1. PACC∼time + covariates×time

2‐4A. PACC∼FC×time + covariates×time

2‐4B. PACC∼FC×Aβ×time + covariates×time

2‐4C. PACC∼FC×Aβ×time + FC×Aβ×time^2^ + covariates×time

Covariates included age, education, APOEe4 status, ICV‐adjusted gray matter volume, treatment group (Solanezumab vs placebo), and head motion. We modeled random effects of intercept and slope for each participant and included scanner as a random effect.

**Result:**

The three‐way quadratic interaction of connectivity and Aβ burden with time was statistically significant for the left FPCN (p‐value corrected for multiple comparisons =.047, Figure 2), with a moderate effect size. That is, stronger left FPCN connectivity was associated with less cognitive decline in those with elevated Aβ. The pattern of effects were similar, but not statistically significant once all covariates were included, for the right FPCN and DMN.

**Conclusion:**

In a very large multi‐site clinical trial dataset, individuals with stronger left FPCN connectivity showed reduced Aβ‐related cognitive decline. This effect was specific to the left‐FPCN, in line with previous cross‐sectional findings. Our finding demonstrates that this protective effect of left FPCN connectivity can be observed in preclinical AD.